# Evaluation of the effects of the Stop GnRH agonist with the letrozole protocol in poor ovarian responders: ABOTH randomized controlled trial

**DOI:** 10.5935/1518-0557.20250036

**Published:** 2025

**Authors:** Nasrin Saharkhiz, Sedighe Hosseini, Mahsa Kazemi, Leila Majdi, Samaneh Esmaeili, Mitra Nemati, Maral Hosseinzadeh, Zahra Zarisfi

**Affiliations:** 1 Department of Obstetrics and Gynecology, School of Medicine, Shahid Beheshti University of Medical Sciences, Tehran, Iran; 2 Department of Biology and Anatomical Sciences, School of Medicine, Shahid Beheshti University of Medical Sciences, Tehran, Iran; 3 Taleghani Hospital Clinical Research Development Unit, School of Medicine, Shahid Beheshti University of Medical Sciences, Tehran, Iran; 4 Preventative Gynecology Research Center, Shahid Beheshti University of Medical Sciences, Tehran, Iran

**Keywords:** poor responders, stop agonist protocol, POSEIDON criteria, ovarian stimulation protocol

## Abstract

**Objective:**

The objective of this study was to evaluate the effect of a stop gonadotropin-releasing hormone (GnRH) agonist with letrozole protocol in improving in vitro fertilization (IVF) cycles in poor ovarian responders (PORs) and to suggest a suitable new ovulation stimulation protocol for this group of infertile women.

**Methods:**

This randomized controlled trial was conducted at the Infertility Center of Taleghani Hospital, Tehran, Iran, from August 2024 to December 2024. The participants were 60 women who fulfilled the POSEIDON Group 4 criteria and had poor ovarian response in their previous IVF cycles. Participants were randomly assigned to the study and control groups and underwent a new IVF cycle. The study group underwent a new cycle with a stop agonist using the letrozole protocol, and the control group underwent the conventional stimulation protocol, which was the same as the previous cycle.

**Results:**

Compared with the conventional protocol, the stop-GnRH agonist and letrozole protocols resulted in a significantly greater number of follicles >13 mm on the day of human chorionic gonadotropin (hCG) administration and a greater number of mature oocytes retrieved, with a significantly greater number of total embryos obtained at days 3 and 5 and a greater number of top-quality embryos. The mean biochemical and clinical pregnancy rates were similar between groups.

**Conclusions:**

The stop-GnRH agonist and letrozole protocol is a short and original protocol that seems to yield better outcomes for patients and may offer promising results for treating POSEIDON Group 4 patients with previous failed IVF.

## INTRODUCTION

Controlled ovarian hyperstimulation (COH) is one of the most critical factors for the success of IVF cycles, and the GnRH antagonist protocol is the most commonly used stimulation protocol in assisted reproduction. However, in a subgroup of “poor responder” patients, there are insufficient mature follicles after gonadotropin stimulation, resulting in cycle cancellation or yield of only a few oocytes (Orvieto *et al.*, 2021).

Poor ovarian responders (PORs) constitute an increasing population, representing 10-24% of women undergoing assisted reproductive technology (ART) (Patrizio *et al.*, 2015; Humaidan *et al.*, 2017). These patients remain a challenge, and several strategies are offered for the treatment of patients with a poor ovarian response to COH (Humaidan *et al.*, 2017; Orvieto *et al.*, 2020). Nevertheless, despite the use of multiple strategies, there is no clear conclusion regarding the ideal COH protocol for these patients (Gonda *et al.*, 2018), and the optimal treatment for poor responders has not yet been established (Garcia-Velasco *et al.*, 2005; Blumenfeld, 2020).

However, in most studies, the conventional antagonist protocol was the preferred treatment for this group of patients (Garcia-Velasco *et al.*, 2005; Humaidan *et al.*, 2017). The conventional IVF cycle using a GnRH antagonist involves several key steps. First, ovarian stimulation is initiated with gonadotropins such as follicle-stimulating hormone (FSH) and/or luteinizing hormone (LH). These hormones are administered to encourage ovaries to produce multiple oocytes. Next, a GnRH antagonist, such as cetrorelix (cetrotide) or ganirelix, is added to prevent premature ovulation. Typically, the administration of GnRH antagonists begins on the sixth day of ovarian stimulation. The final step in this process is the trigger shot, which usually contains human chorionic gonadotropin (hCG) and is used to mature oocytes. This trigger shot is given when the leading follicle reaches a specific size, typically approximately 18 mm (Garcia-Velasco *et al.*, 2005; Blumenfeld, 2020). This structured approach aims to optimize the chances of successful oocyte retrieval during the IVF process (Humaidan *et al.*, 2017; Orvieto *et al.*, 2020).

One recent study demonstrated that combining the stop GnRH agonist protocol with the GnRH antagonist protocol in PORs that previously failed several IVF treatment cycles resulted in a significantly greater number of oocytes retrieved and top-quality embryos (TQEs), with a reasonable clinical pregnancy rate (Gonda *et al.*, 2018).

Based on previous studies, letrozole functions as an aromatase inhibitor, and by inhibiting the synthesis of estradiol, it reduces negative feedback and increases endogenous gonadotropin secretion (Garcia-Velasco *et al.*, 2005; Orvieto *et al.*, 2021). Letrozole has been shown to reduce the dosage of FSH required for COH, while also improving the ovarian response in individuals identified as poor responders, and is offered as a useful co-treatment for this frustrating group of patients (Garcia-Velasco *et al.*, 2005; Davar *et al.*, 2010). Moreover, letrozole increases intraovarian androgen activity and enhances the expression of FSH receptors in granulosa cells, improving ovarian response to FSH in poor responders, resulting in a greater number of retrieved oocytes and a greater implantation rate in patients whose IVF cycle has been canceled (Davar *et al.*, 2010; Orvieto *et al.*, 2021). Co-administration of letrozole and GnRH agonists for COH in poor responders may increase the pregnancy rate in ART cycles (Garcia-Velasco *et al.*, 2005); therefore, we designed a randomized controlled trial to evaluate the GnRH antagonist with letrozole protocols in poor responders undergoing IVF cycles.

GnRH antagonists and agonists are equally recommended for poor responders, but their use in ART remains a challenge and the efficiency of their stimulation protocol is still being discussed (Garcia-Velasco *et al.*, 2005; Poseidon Group *et al.*, 2016; Lambalk *et al.*, 2017). LH secretion was immediately blocked using a GnRH antagonist. GnRH antagonists prevent the LH surge from occurring within a few hours, which is a common cause of cancellation in patients with poor ovarian response but does not result in early folliculogenesis inhibition, which is critical for patients with a limited number of follicles (Garcia-Velasco *et al.*, 2005; Blumenfeld, 2020).

The POSEIDON (Patient-Oriented Strategies Encompassing Individualized Oocyte Number) classification system was developed to stratify POR patients and guide tailored treatment approaches (Humaidan *et al.*, 2016). POSEIDON’s classification enables the definition of low-prognosis patients regarding their ability to produce at least one euploid embryo, dividing them into four subgroups according to qualitative and quantitative parameters (Humaidan *et al.*, 2016; Poseidon Group *et al.*, 2016). In 2016, the POSEIDON group introduced new criteria for the classification of poor responders, and the most frustrating subgroup of these patients was defined as POSEIDON group 4 (patients aged >35years, AFC <5, and anti-Müllerian hormone [AMH] <1.2ng/mL) (Humaidan *et al.*, 2016; Poseidon Group *et al.*, 2016). This stratification attempts to differentiate between relevant subpopulations of poor responders, for whom specific interventions might be beneficial in more tailored and efficient care, facilitating the evaluation of strategies that could generate higher success in ART for particular subgroups of patients in clinical trials (Leijdekkers *et al.*, 2019).

Schachter *et al.* (2001) and Hazout *et al.* (1993) hypothesized that POR benefits from double stimulation (flare-up effect, then gonadotropins) associated with a less strenuous blockage (discontinuation of GnRH agonist) to favor follicular recruitment to obtain a better ovarian response and produce more oocytes and embryos, including more usable embryos, increasing the likelihood of ongoing pregnancies in patients with a poor prognosis.

Therefore, based on previous observations, we offered POSEIDON Group 4 patients a novel protocol, combining the stop GnRH agonist protocol with letrozole priming, to improve the follicular response and sensitivity to FSH. This new protocol may be an appropriate treatment strategy for patients with a poor ovarian response.

We proposed to our poor responder patients the stop GnRH agonist and letrozole protocol, which uses a GnRH agonist for its flare-up effect in the luteal phase of the cycle and then stops, enabling pituitary desensitization to prevent a premature LH surge associated with controlled ovarian stimulation with gonadotropins at the maximum dosage (300 IU/day) (Ngwenya *et al.*, 2024). This protocol causes less blockage by discontinuing the GnRH agonist, and is associated with better follicular recruitment and ovarian response.

This protocol combines downregulation with a GnRH agonist starting in the luteal phase, cessation of GnRH agonist therapy with the onset of menstruation, and high-dose gonadotropin administration. This short-term ovarian suppression, which began in the luteal phase and was discontinued with the onset of menses, yielded favorable pregnancy results in PORs. These procedures eliminate excessive ovarian suppression while benefiting from the additional gonadotropin stimulus provided by the agonistic effect of the GnRH agonist (Bavarsadkarimi *et al.*, 2022).

This study was performed to evaluate whether, in poor responders, the stop GnRH agonist and letrozole protocol allows for a greater number of mature oocytes retrieved (primary outcome), total number of embryos observed on days 3 and 5, number of TQEs, chemical pregnancy rate, and clinical pregnancy rate (secondary outcomes) compared to the previous IVF attempt (conventional protocol).

Our goal was to conduct a randomized controlled trial (RCT) to determine whether the two regimens, the GnRH agonist with the letrozole regimen and the antagonist regimen, differ in their effectiveness in women with POR diagnosed according to the POSEIDON criteria.

## MATERIALS AND METHODS

We conducted a randomized controlled trial of poor responders at the Infertility Center of Taleghani Hospital, Tehran, Iran, from August 2024 to December 2024. The inclusion criteria were poor ovarian response to conventional GnRH antagonist IVF/intracytoplasmic sperm injection (ICSI) cycles and fulfillment of the POSEIDON Group 4 criteria.

Patients included in the study were ≥35 and <42 years old and had poor prognoses according to the POSEIDON stratification. The recorded patient data included age, body mass index (BMI), AMH level, AFC, and infertility duration. The measured parameters included duration of stimulation, total dose of gonadotropin used, number of follicles > 13 mm, administration on the day of hCG, number of oocytes retrieved, number of total embryos, number of TQEs, chemical pregnancy rate, and clinical pregnancy rate. The primary outcome was the number of mature oocytes retrieved, and the secondary outcomes were the number of total embryos observed on days 3 and 5, number of TQEs, chemical pregnancy rate, and clinical pregnancy rate.

Patients were excluded from the study if they met at least one of the following criteria: 1) the presence of a clinically significant systemic disease, diabetes mellitus; 2) PCOS, hyperprolactinemia, or any other endocrine disorder; 3) submucosal polyp, leiomyoma, or uterine septum; or 3) severe male factor or azoospermia.

### Randomization and blinding

We included women whose cycles were terminated because three or fewer mature follicles or eggs were retrieved following maximal stimulation with at least 300 IU of gonadotropin per day. The participants were randomly assigned, and 60 women were randomized into two groups. The study group (n=30) underwent a subsequent COH via the stop-GnRH agonist and letrozole protocol within 2 months of the previous failed conventional IVF/ICSI cycle, and the control group (n=30) underwent a conventional antagonist cycle similar to the previous cycle. The embryologist who assisted in the procedure was blinded to the treatment allocation. The statistician was blinded to the allocated treatment while analyzing the data.

In the conventional antagonist cycle (control group), gonadotropins (Cinnal-f, follitropin alfa; CinnaGen, Iran) were administered on days 2-3 of the menstrual cycle, with a minimal daily dose of 150 IU, depending on the patient’s age and ovarian reserve. In some cases, this was followed by highly purified HMG (PD-HOMOG, Pooyesh Darou, Iran), with a minimal daily dose of 75 IU. Continuous doses of drugs were adjusted according to vaginal ultrasound measurements of follicular diameter every 2 or 3 days. A subcutaneous GnRH antagonist, Cetronax (Cetrorelix Acetate; Ronak Pharma, Iran), 250 µg/day, was started when the follicle size reached a diameter of ≥14 mm.

Gonadotropin and cetrotide injections were continued until triggering day. Ovulation and final follicular maturation were triggered using 10,000 IU hCG (PDPREG, Pooyesh Darou, Iran) when the leading follicles reached a mean diameter of 18 mm. Transvaginal oocyte retrieval was performed 36 hours later using a double-lumen needle. ICSI was performed, as appropriate. Cycle cancellation was considered when fewer than two follicles with normal growth patterns were noted.

**Figure 1:**
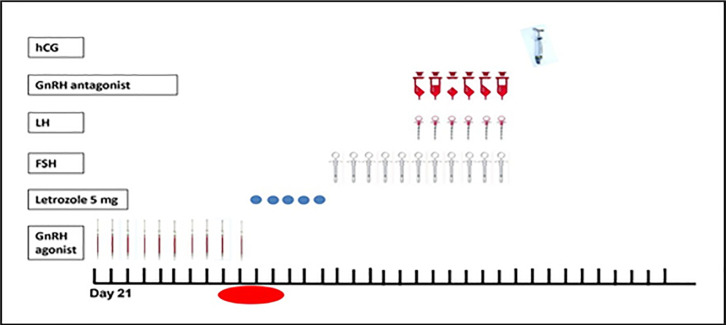
The GnRH agonist and letrozole regimens were discontinued. (hCG: human chorionic gonadotropin; LH: luteinizing hormone; FSH: follicle-stimulating hormone)

In the study protocol, patients received a daily GnRH agonist, triptorelin (Variopeptyl; Varian Pharmed, Iran), 0.1 mg/day, starting in the mid-luteal phase and discontinued at the onset of menses and after confirmation of downregulation by vaginal ultrasound measurements. In the following 5 days, the patients received letrozole (5 mg/day) and were then stimulated with gonadotropins (Figure 1). Once the leading follicle had reached a size of ≥14 mm, co-treatment with 250 µg/day GnRH antagonist (Cetrorelix Acetate; Ronak Pharma, Iran) was initiated and continued until the day of hCG administration. Final follicular maturation was triggered using 10,000 IU (hCG chorionic gonadotropin hCG (PDPREG, Pooyesh Darou, Iran) when the leading follicles reached a mean diameter of 18 mm. Oocytes were retrieved under transvaginal ultrasound guidance 36 hours after the hCG trigger. Intracytoplasmic sperm injection was performed as described for other groups. Cycle cancellation was considered when fewer than two follicles with normal growth patterns were noted.

The luteal phase was supported similarly, and all patients were administered a vaginal progesterone suppository (Actogest, Actovere) at 400mg/day and intramuscular progesterone (Aburaihan, Tehran, Iran) at 50mg/day, starting on the day of oocyte retrieval and continuing until a negative pregnancy test or 8 weeks of gestation.

Embryo classification was based on individual embryo scoring parameters according to pre-established definitions (Ziebe *et al.*, 2007). At D3, embryo morphology was graded using a standard system, including the number, size, and uniformity of blastomeres, degree of fragmentation, and presence of multinucleated blastomeres. A TQE was defined as seven or more blastomeres on day 3, equally sized blastomeres, and <15% fragmentation. All the other characteristics were associated with poor embryo quality. At D5, blastocyst morphology was evaluated according to the Gardner and Schoolcraft grading system (Naik *et al.*, 2024). Thus, usable blastocysts were defined as full (grade 3), expanded (grade 4), partially hatched (grade 5), or fully hatched (grade 6) blastocysts with at least grade B trophectodermal quality.

The usable embryos were freshly transferred. Depending on the number and quality of available embryos and the patient’s age, embryo transfer (ET) was performed under transabdominal ultrasound guidance 3 or 5 days after oocyte retrieval, depending on the number and quality of available embryos and patient age. Therefore, fresh embryo transfers were performed at either the cleavage (D3) or the blastocyst stage (D5). Surplus-usable embryos (D3 or D5) were cryopreserved for subsequent transfers. The embryo transfer strategy was determined by a multidisciplinary team, and a maximum of two embryos were replaced.

To confirm pregnancy, serum beta-hCG levels were measured approximately 2 weeks after ultrasound-guided embryo transfer. Following a positive pregnancy test, an ultrasound scan was performed to confirm the continuation of the pregnancy at 6-8 weeks of gestation. Biochemical pregnancy was characterized by the absence of an identifiable pregnancy on ultrasound examination despite a positive blood hCG pregnancy test, and clinical pregnancy was confirmed if a fetal heartbeat could be observed by transvaginal ultrasound.

Data on patient age, infertility-related variables, ovarian stimulation characteristics (dose of gonadotropins and duration of stimulation), number of follicles >13 in diameter on the day of hCG administration, number of oocytes retrieved, total number of embryos, number of TQEs, pregnancy rates, and cancellation rates were collected and compared between the two groups (conventional group *vs*. study group).

### Outcomes and sample size calculation

The main outcome measure was the total number of mature oocytes obtained from the poor responders after ovarian stimulation. The secondary outcome measures were the total number of embryos at days 3 and 5, number of TQEs, biochemical pregnancy rate, and clinical pregnancy rate.

Sample size calculation was performed to ensure adequate statistical power to detect differences in the primary outcome. Based on previous studies (Garcia-Velasco *et al.*, 2005; Orvieto *et al.*, 2021), we assumed a mean difference of 2 mature oocytes retrieved between the stop-GnRH agonist with letrozole protocol and the conventional antagonist protocol, with a standard deviation of 2.5. Using a two-sided significance level (α) of 0.05 and a power of 80%, a minimum of 26 participants per group was required. Accounting for potential dropouts (approximately 15%), we enrolled 30 participants per group, totaling 60 participants. This calculation was performed using G*Power software (version 3.1).

### Statistical analysis

We used a two-sample t-test with degrees of freedom (df) = 58 to calculate the p-values based on the t-statistics provided. For continuous variables, we used a two-sample t-test (or Welch’s t-test, where degrees of freedom varied). For proportions, we used Fisher’s exact test based on assumed counts derived from the percentages and sample size (n=30 per group).

The results and quantitative variables are presented as mean±standard deviation (SDs). Statistical significance was accepted at *p*<0.05. SPSS version 15.0 (SPSS Inc., Chicago, IL, USA) was used for the data analysis.

## RESULTS

Sixty POR women with previous failed IVF cycles were randomly assigned to one of the two study arms, with 30 women in each group. A new ovarian stimulation cycle was performed: 30 women underwent the stop-GnRH agonist and letrozole protocol, and the other 30 women were stimulated by a conventional GnRH antagonist regimen. The baseline characteristics of the two groups were comparable, including age, BMI, AMH level, AFC, duration of infertility, and number of previous failed IVF cycles. Table 1 shows the baseline characteristics of women who were randomly assigned to one of the two regimens. This table compares the “stop GnRH agonist” group (n=30) and the “GnRH antagonist” group (n=30) for five continuous variables. I used a two-sample t-test with degrees of freedom (df) = 58 to calculate the p-values based on the provided t-statistics.

**Table 1:** Baseline characteristics of women who underwent the stop GnRH agonist and antagonist protocols.

	All (n=60)	Stop GnRH agonist (n=30)	GnRH antagonist (n=30)	p-value
Age	38.4± 2.8	38.3±2.3	38.5±3.4	0.790
BMI (means±SDs)	25.7±4.6	25.6±4.1	25.8±5.1	0.868
AMH (ng/ml)	0.76±0.75	0.71±0.7	0.82±0.6	0.516
AFC (means±SDs)	4.1±1.8	4.1±1.4	4.2±2.3	0.840
Infertility duration (year) (means±SDs)	3.8±6.2	4.1±5.6	3.5±6.8	0.710

The overall mean age of the participants was 38.4±2.8 years, the mean BMI was 25.7±4.6, the mean basal serum AMH level was 0.7±0.75 ng/mL, the mean AFC was 3.8±6.2, and the mean infertility duration was 3.8±6.2. There were no significant differences among the groups concerning women’s age (38.3 years *vs*. 38.5 years), body mass index (25.6 kg/m^2^ *vs*. 25.8 kg/m^2^), serum AMH level (0.71 ng/ml *vs*. 0.82 ng/ml), AFC (4.1 *vs*. 4.4), or duration of infertility (4.1 years *vs*. 3.5 years).

The treatment data and stimulation characteristics of the IVF cycles in the two groups were evaluated, and the results are shown in Table 2. The measured parameters included the duration of stimulation, total dose of gonadotropin used, number of follicles >13mm administered on the day of hCG administration, number of oocytes retrieved, total number of embryos, number of TQEs, biochemical pregnancy rate, clinical pregnancy rate, and cancellation rate. This table includes continuous variables and proportions. For continuous variables, I used the two-sample t-test (or Welch’s t-test, where degrees of freedom varied). For proportions, I used Fisher’s exact test based on assumed counts derived from the percentages and sample size (n=30 per group).

**Table 2:** Treatment data and stimulation characteristics of the IVF cycles in the 2 groups.

	All (n=60)	Stop GnRH agonist (n=30)	GnRH antagonist (n=30)	p-value
Duration of stimulation (day) (means±SDs)	11.0±5.2	12.9±6.2	9.1±2.9	0.004
Total dose of gonadotropin used (IU) (means±SDs)	3076.8±465.1	3321.2±152.8	2832.4±541.4	<0.001
Number of follicles >13 mm on day of hCG (means±SDs)	2.8±0.7	3.4±0.2	2.2±0.5	<0.001
Number of mature oocytes retrieved (means±SDs)	2.3±2.5	3.19±2.62	1.34±2.12	0.004
Number of total embryos				
(means±SD)	2.4±1.3	2.6±1.6	2.1±0.7	0.124
Number of TQE (means±SDs)	1.5±1.5	1.62±1.67	1.46±1.42	0.691
Biochemical Pregnancy Rate (%)	6.15	6.32	5.98	1.000
Clinical Pregnancy Rate (%)	4.9	4.9	4.9	1.000
Total Cancellation Rate (%)	2	2	2	1.000

TQE: top-quality embryo; IVF: *in vitro* fertilization.

As expected, patients receiving the stop GnRH agonist protocol required significantly higher doses and longer durations of gonadotropin stimulation. The duration of stimulation (12.9±6.2 *vs*. 9.1±2.9 days, respectively) and the total dose of gonadotropins (3321.2±152.8 *vs*. 2832.4±541.4 IU, respectively) differed significantly between the two groups. These parameters were significantly greater in the stop-GnRH agonist regimens than in the routine antagonist regimens (*p*<0.05).

The patients in the study group also presented significantly greater numbers of follicles >13 mm in diameter on the day of hCG administration (3.4±0.2 *vs*. 2.2±0.5, *p*<0.05, respectively), significantly more oocytes retrieved (3.19±2.62 *vs*. 1.34±2.12, *p*<0.05, respectively), and a nonsignificantly greater number of total embryos and a greater number of TQEs. Therefore, the number of mature oocytes retrieved was significantly greater in the stop-GnRH agonist and letrozole protocols than in the conventional antagonist protocol, and there was no significant difference in the mean number of total embryos or TQEs.

The biochemical pregnancy rate was 6.32% with the stop-GnRH agonist and letrozole protocol and 5.98% with the antagonist protocol, with no statistically significant difference (*p*=1.00). The clinical pregnancy rate was 4.9% with the stop-GnRH agonist and letrozole protocol and 4.9% with the antagonist regimen, with no statistically significant difference (*p*=1.00). Therefore, the biochemical and clinical pregnancy rates were similar in both groups and did not differ significantly between the two regimens.

## DISCUSSION

While assisted reproductive strategies have become more advanced with excessive achievement fees in terms of pregnancy and live birth fees, poor responders remain a research assignment for assisted reproductive experts. A recent proof-of-concept study of Poseidon group 4 POR patients offered combined stop GnRH agonist and letrozole priming (for 5-7 days from confirmation of downregulation until the start of OS) combined with pretreatment pituitary suppression. Patients had a significantly greater number of follicles >13mm on the day of hCG administration and a greater number of oocytes retrieved, with significantly greater numbers of TQEs and a reasonable clinical pregnancy rate. Maintaining pituitary suppression after downregulation provides a “5-7 day pause” for letrozole priming (Orvieto *et al.*, 2021). This pause allows the development of additional follicular waves, while enabling letrozole to increase intrafollicular androgen levels (Garcia-Velasco *et al.*, 2005) and augment FSH receptor expression in granulosa cells, with a consequent increase in FSH-sensitive antral follicles (Davar *et al.*, 2010). The purpose of this study was to compare the efficacy of conventional GnRH agonist, letrozole, and GnRH antagonist regimens in infertile women who had a poor response to ovarian stimulation during IVF. The primary outcome of this study was the number of mature oocytes retrieved.

Notably, the stop-GnRH agonist with letrozole group required a significantly higher total gonadotropin dose (3321.2±152.8 IU *vs*. 2832.4±541.4 IU, *p*<0.001) and longer stimulation duration (12.9±6.2 days *vs*. 9.1±2.9 days, *p*=0.004) compared to the conventional group. This raises the question of whether the increased gonadotropin dose could explain the greater number of mature oocytes retrieved (3.19±2.62 *vs*. 1.34±2.12, *p*=0.004). While higher doses might enhance follicular recruitment, poor responders often exhibit diminished ovarian sensitivity, suggesting that the protocol itself, rather than the dose alone, contributes significantly to the outcome. Literature indicates that beyond a certain threshold (e.g., 300 IU/day), additional gonadotropin dosing yields limited returns in this population (Blumenfeld, 2020; Gerber *et al.*, 2020). To explore this further, a sub-analysis matching participants by total gonadotropin dose was considered. However, due to the small sample size (n=30 per group) and significant variability in doses (range: 152.8-541.4 IU SD), insufficient overlap precluded a meaningful matched comparison. This limitation highlights the need for larger studies to isolate the protocol’s effect independent of dose.

The study revealed that GnRH agonists typically led to increased gonadotropin consumption and a prolonged period of stimulation at the same time as in the assessment of antagonist regimens. In the present study, POSEIDON Group 4 PORs (patients aged >35years with poor ovarian reserve and an AFC <5), the stop GnRH agonist and letrozole cycle provided a significantly greater number of follicles, more retrieved mature oocytes, and non-significantly more total embryos and TQEs for transfer than the conventional antagonist cycle. The total cancellation rate was similar in both groups. This prospective study revealed that the stop-GnRH agonist and letrozole stimulation protocol is a short and original protocol that strengthens the therapeutic arsenal of poor responders, which may offer promising results for patients with a poor prognosis and a record of failed IVF. The long GnRH agonist protocol pretreatment results in a better-synchronized response; however, continuing the GnRH agonist during COH is often associated with a significant increase in the required dose of gonadotropins, and its cessation might improve the ovarian response and prevent the need to increase the daily dose of gonadotropin (Orvieto & Patrizio, 2013). Maintaining pituitary suppression provides a “5-day pause,” allowing the development of additional follicular waves while enabling letrozole priming. The consequent increase in intrafollicular androgen levels may augment FSH receptor expression in granulosa cells, with a consequent increase in the number of FSH-sensitive antral follicles (Ubaldi *et al.*, 2016). The stopped GnRH agonist, together with the GnRH antagonist, provides immediate LH suppression, eliminating the premature LH surge, and might improve the quality of the embryos generated (Haas *et al.*, 2019). In a previous meta-evaluation of 14 research studies performed with the aid of Danhua Pu *et al*., a shorter duration of stimulation with GnRH antagonists was also observed. In the same study, no significant difference in the number of retrieved oocytes or mature oocytes was found, which is in agreement with our findings. Furthermore, these authors reported no significant difference in clinical pregnancy rates, as we found in the present study (Pu *et al.*, 2011). Interestingly, a recent meta-analysis of six poor responder-related studies revealed no evidence of a difference in the clinical pregnancy rate (Lambalk *et al.*, 2017). Our study revealed the same clinical pregnancy rate with the use of a short GnRH agonist compared to a GnRH antagonist. In addition, there were no differences in oocyte yield in the aforementioned meta-analysis, as found in the present study (Lambalk *et al.*, 2017). However, Pu *et al*. combined GnRH agonist studies into one group, regardless of whether it was a longor short-agonist regimen (Pu *et al.*, 2011). Although the clinician performing the egg collection procedure and the embryologist assessing the number of eggs were blinded to the study protocol, the clinicians involved in the decision-making for hCG administration to induce ovulation were not involved, which is a study weakness. Although most women had a long GnRH agonist regimen in the previous cycle, the randomization process was not stratified by the previous regimen, which could be a confounder. Based on preceding RCTs, GnRH agonist protocols appear to have an extra edge in terms of medical pregnancy and cycle cancellation prices than GnRH antagonist protocols, even though in a single middle RCT, the GnRH antagonist protocol was related to better pregnancy prices compared to GnRH agonist regimen (Siristatidis *et al.*, 2016; Naik *et al.*, 2024). For poor responder subgroup management, the European Society of Human Reproduction and Embryology (ESHRE) Guideline Group on Ovarian Stimulation currently recommends both GnRH antagonists and GnRH agonists (Ovarian Stimulation *et al.*, 2020). In Loutradis *et al.* (2004) and Badawy *et al.* (2012) reviews, for poor responders, GnRH agonist flare-ups and long agonist protocols did not seem to be as advantageous as a reduction in GnRH agonist doses, “stop” protocols, or microdose GnRH agonist flare-regimens. These regimens all appear to improve outcomes, although the benefit of one approach over another has not been convincingly established, with no difference between their outcomes (Badawy *et al.*, 2012).

A more recent RCT revealed that microdose flare-up seems to be superior to flare-up, with a significantly greater LBR (*p*=0.036) (Ghaffari *et al.*, 2020) but similar efficacy to that of the GnRH antagonist protocol (Kahraman *et al.*, 2009; Davar *et al.*, 2010).

The use of GnRH agonists during COS in long protocols may lead to a poor ovarian response owing to intense endogenous FSH suppression and the possible local inhibitory effect of GnRH agonists on the ovaries (Hong *et al.*, 2019; Yildiz *et al.*, 2020). The stop GnRH agonist and letrozole protocols may overcome these adverse effects by enhancing the release of early follicular phase FSH with a flare-up effect, intensifying the effects of exogenous gonadotropins. The advantage of the stop-GnRH agonist and letrozole protocols over the long protocol is the shorter duration of stimulation, which could favor better compliance and tolerance.

Short-term use of GnRH agonists (7 days) does not profoundly inhibit ovarian response through ovarian GnRH receptors, while sufficiently inhibiting premature LH surges (Ubaldi *et al.*, 2016; Blumenfeld, 2020). In the stopped GnRH agonist and letrozole groups, no cancellations were observed due to premature LH surge or ovulation in the 7 days following the discontinuation of GnRH agonist.

After stopping the GnRH agonist (5-day course), endogenous GnRH activity appeared to be suppressed for at least 7 days because the pituitary is in a refractory state of LH secretion, as found in (Cedrin-Durnerin *et al.*, 2000), which revealed decreased LH concentrations after early discontinuation of GnRH agonist administration compared with a long agonist protocol. Indeed, hypophyseal desensitization is related to GnRH receptor reduction, leading to a progressive reduction in gonadotropin synthesis that persists for several days (Blumenfeld, 2020; Bavarsadkarimi *et al.*, 2022).

We found that the mean number of usable embryos was greater in the stop-GnRH agonist and letrozole protocols, but the difference was not statistically significant. The number of cumulative ETs in the stop-GnRH agonist and letrozole groups was greater (54 *vs*. 42), but the difference was not statistically significant (*p*=0.124). Twelve surplus embryos are waiting for ET (mainly because of an ongoing pregnancy); therefore, the number of cumulative ETs would probably be significant if all the embryos were transferred, with potentially more pregnancies. Schachter *et al*. also reported significantly more cleaving embryos with improved morphology after discontinuing the GnRH agonist protocol than after the long agonist protocol (Schachter *et al.*, 2001).

The freeze-all rate was significantly higher in the stop GnRH agonist and letrozole protocols, mostly because of prolonged stimulation, an indication that prolonged stimulation is associated with decreased ART success because of an impaired endometrium for implantation (except for PCOS) (Pereira *et al.*, 2017). However, a recent study revealed that the total dose of gonadotropin affects LBR in fresh cycles (Gerber *et al.*, 2020). Therefore, a freeze-all strategy involving only the total gonadotropin dose (>5000 IU) would be more appropriate.

The miscarriage rate (MR) in both groups was particularly low, probably because of the small size of our population and the selection of our population with previous failed IVF cycles (no pregnancy, biochemical pregnancies, or miscarriages). There is no reason to believe that the stop GnRH agonist and letrozole protocol could reduce miscarriage by increasing the ploidy rate, because recent studies have shown that ovarian stimulation does not affect the risk of aneuploidy (Hong *et al.*, 2019).

A limitation of our analysis was the small sample size. However, based on our patient selection process, only patients who fulfilled the inclusion criteria were enrolled, which considerably decreased the likelihood of selection bias.

Another limitation is that cycle-to-cycle variation exists in the ovarian response and that the growth and steroidogenic characteristics of antral cohorts in response to exogenous FSH may vary from one cycle to another (e.g., the expression and sensitivity of FSH receptors in granulosa cells) (Yildiz *et al.*, 2020). However, cycle-to-cycle heterogeneity was probably similar in both groups. The overall limitations of the aforementioned studies are the relatively small sample size, the use of more medications (which might be more expensive), and their complexity (which might be more confusing for patients).

## CONCLUSION

We chose to focus on a specific population among all poor responders (POSEIDON Group 4) with three or fewer oocytes following conventional COH for IVF with high daily dose gonadotropins (>300 IU) because these patients are the most challenging.

The stop-GnRH agonist and letrozole protocols relatively enhance cycle programming and better follicular synchronization and, in some cases, may offer promising results (more mature oocytes and embryos) in poor responders and Poseidon group 4 patients; however, more randomized controlled studies are needed to strengthen this concept. These results must be confirmed by a large prospective study evaluating the live birth rate after this protocol versus the standard protocol.

In conclusion, based on the current study and in terms of effectiveness, the stop GnRH agonist and letrozole protocol could be chosen as a first-choice approach while keeping in mind the greater duration of stimulation typically required in such a protocol.

### Ethics approval and consent to participate

We confirm that we have read the journal’s position on issues involved in ethical publication and affirm that this report is consistent with these guidelines. This study was approved by the Ethics Committee of Shahid Beheshti University of Medical Sciences (IR.SBMU.RETECH.REC.1403.236). All participants provided written informed consent before participation and were informed of the study protocol.
